# Dark triad personality traits and online impulsive buying: the mediating role of social media addiction

**DOI:** 10.3389/fpsyg.2026.1879807

**Published:** 2026-07-24

**Authors:** Ümit Doğrul, Selen Salman

**Affiliations:** 1 Department of Business Administration, Faculty of Economics and Administrative Sciences, Mersin University, Mersin, Türkiye; 2 Institute of Social Sciences, Mersin University, Mersin, Türkiye

**Keywords:** consumer psychology, dark triad personality, latent state-trait theory, online impulsive buying, social media addiction

## Abstract

**Objective:**

The rapid growth of e-commerce has made online impulsive buying (OIB) an increasingly significant consumer behavior. However, the psychological processes underlying this behavior—particularly the role of individual differences—remain inadequately explained. This study adopts the Latent State–Trait (LST) approach as an interpretive conceptual framework to examine the relationships among the Dark Triad personality traits (DTP) (narcissism, Machiavellianism, and psychopathy), social media addiction (SMA), and online impulsive buying.

**Method:**

Cross-sectional research data were collected from 413 participants using validated scales measuring the Dark Triad personality traits, social media addiction and online impulsive buying behavior. The proposed research model was tested using covariance-based structural equation modeling (CB-SEM); indirect effects were examined using bootstrap confidence intervals.

**Findings:**

All three dimensions of the Dark Triad personality traits were positively associated with social media addiction (β = 0.24-0.32), and social media addiction was positively associated with online impulsive buying behavior (β = 0.61). Furthermore, social media addiction exhibited significant indirect effects on the relationships between narcissism (β = 0.194, 95% CI [0.138, 0.261]), Machiavellianism (β = 0.183, 95% CI [0.127, 0.245]), and psychopathy (β = 0.217, 95% CI [0.154, 0.286]) and online impulsive buying, as none of the bootstrap 95% confidence intervals included zero.

**Conclusion:**

The findings are consistent with the proposed trait–state–behavior framework and suggest that social media addiction is significantly associated with the relationship between Dark Triad personality traits and online impulsive buying. As the study is based on cross-sectional data, the findings should be interpreted as associations rather than evidence of temporal or causal relationships. By adopting the LST perspective as a conceptual framework, this study contributes to a broader understanding of the relationships among personality traits, social media addiction, and online impulsive buying.

## 1 Introduction

Recent developments in digital technologies have contributed to the rapid growth of e-commerce and changed consumers' shopping habits (Ngo et al., 2025). Today, online shopping has become a part of everyday life for many consumers. Personalized product recommendations, targeted advertisements, and quick access to products have a greater influence on consumers' purchase decisions than before (Kathuria and Bakshi, 2025). Along with these changes, online impulsive buying (OIB) has also become more common. For example, Amra and Elma (2026) reported that 91% of consumers had made at least one impulsive purchase, 84% of online shoppers had bought products they had not planned to purchase, and in some sectors these purchases accounted for around 60–70% of total sales. These findings show that OIB has become an important part of today's e-commerce.

However, OIB should not be seen simply as a behavior that provides immediate pleasure. Research shows that unplanned purchases may provide short-term satisfaction. At the same time, they may also result in negative outcomes such as guilt, regret, and financial problems (Wang et al., 2022). Therefore, it is not enough to explain OIB only by the opportunities provided by online shopping environments. Consumers shopping in the same online environment do not always behave in the same way, even when they are exposed to similar stimuli. Some consumers are more easily influenced by these stimuli, whereas others are able to control their purchase decisions. This suggests that OIB is associated not only with environmental factors but also with individuals' relatively stable psychological characteristics.

At this point, personality traits play an important role in explaining why individuals behave differently (Mount et al., 2005). In particular, the “Dark Triad” personality traits (DTP)—comprising narcissism, Machiavellianism, and psychopathy—are receiving increasing attention in explaining consumer behavior. Previous studies have examined these personality traits in relation to panic buying (Yousaf et al., 2022; Piper et al., 2024), purchase intention (Koay and Lok, 2025), consumption of luxury and counterfeit goods (Ahn, 2023; Razmus et al., 2023), unethical consumption behaviors (Egan et al., 2015; Harrison et al., 2018), and word-of-mouth communication (Wien et al., 2024).

However, existing research has primarily examined the direct relationships between the DTP and consumer behavior (Jones and Paulhus, 2011; Yousaf et al., 2022), while giving comparatively less attention to the psychological factors through which these relatively stable personality traits are associated with consumer behavior. Although several mediating mechanisms have been proposed, including consumer self-confidence and aggressive interpersonal orientation (Koay and Lok, 2025), moral disengagement (Ahn, 2023), and the need for approval (Akoglu and Cesur, 2025), these studies typically examine isolated psychological mechanisms. As a result, the literature still lacks a broader conceptual framework that jointly considers relatively stable personality traits, context-sensitive psychological factors, and OIB within digital environments.

The importance of individual differences becomes even more pronounced in digital environments saturated with marketing stimuli. Social media platforms, in particular, constantly expose users to advertisements, social feedback, social comparisons, and personalized content. This situation is associated with an increase in impulsive purchasing tendencies, as well as a weakening of cognitive control and a strengthening of impulsive behaviors (Zheng et al., 2019; Chen et al., 2021). Furthermore, intensive and prolonged use of social media is linked to SMA. Therefore, SMA may represent an important psychological factor associated with the relationship between personality traits and OIB. In other words, SMA can be viewed as a context-sensitive psychological factor associated with the interaction between relatively stable personality traits and characteristics of the digital environment.

Unlike most existing studies, which largely rely on direct effect models, this research adopts a process-oriented conceptual perspective to better explain the relationships among the DTP, SMA, and OIB. Accordingly, these relationships are interpreted within a conceptual trait–state–behavior framework. In this study, the LST approach (Steyer et al., 2015) was used not as an empirically tested LST model, but as a conceptual framework to help interpret these relationships. Within this framework, the DTP are considered relatively stable, trait-like constructs, while SMA is viewed as a context-sensitive, state-like psychological factor. OIB, meanwhile, was treated as a behavioral outcome within this conceptual framework. However, since this study is based on cross-sectional data collected at a single point in time, the proposed trait–state–behavior sequence should be interpreted as a theoretical representation of the observed relationships rather than as empirical evidence of temporal or causal processes.

The present study contributes to the literature in two ways. First, it goes beyond the direct-effect approach that dominates the current literature by examining the relationships among the DTP, SMA, and OIB within a process-oriented conceptual framework. Second, rather than empirically testing the LST model, it demonstrates that this perspective provides a useful conceptual framework for interpreting these relationships in the context of digital consumption. Thus, the study offers a broader conceptual perspective for understanding the relationships among individual tendencies, SMA, and OIB.

## 2 Theoretical background

### 2.1 Latent state-trait theory

The Latent State–Trait (LST) theory suggests that both individual traits and situational effects should be considered when explaining psychological outcomes (Steyer et al., 2015). According to the theory, individual differences come from two main sources. The first is relatively stable personal traits (trait). The second is temporary effects (state), which can change depending on the situation and context (Setyadi et al., 2024). Therefore, observed psychological constructs are shaped not only by individual traits but also by situational effects and the interaction between the two (Shefi, 2025). This perspective implies that psychological phenomena are shaped not only by individual traits but also by the conditions under which those traits are expressed (Liu and Cole, 2025). Accordingly, LST theory argues that both personal characteristics and situational influences should be considered when seeking to understand why behavior varies across contexts (Kaur and Sharma, 2024).

This perspective is particularly relevant for explaining OIB, a behavior that develops within digital environments where individual characteristics and environmental conditions are closely intertwined (Chan et al., 2017; Zafar et al., 2023). In the present study, the DTP are conceptualized as relatively stable dispositions reflecting enduring psychological tendencies (Paulhus and Williams, 2002). SMA, by comparison, is viewed as a context-sensitive psychological process that may emerge through the interaction between these individual characteristics and the highly stimulating nature of social media environments (Kircaburun et al., 2019; Demircioglu and Göncü Köse, 2021; Liao et al., 2025).

This study adopts the LST approach not as an implicit state–trait model tested empirically, but as a conceptual interpretative framework. Accordingly, the proposed trait–state–behavior sequence aims to theoretically organize the relationships between the DTP, SMA and OIB. Within this framework, the DTP are regarded as relatively stable trait-like constructs; SMA as a context-sensitive state-like psychological process; and OIB as the behavioral outcome of these relationships. The present study does not claim to modify, reinterpret or extend the classical LST theory. Rather, the LST approach is used as a conceptual lens to help interpret the relationships between relatively stable personality traits, context-sensitive psychological processes and online consumer behavior. Furthermore, as the study is based on cross-sectional data collected at a single point in time, this conceptual framework should be regarded not as providing empirical evidence regarding temporal or causal processes, but rather as a theoretical interpretation of the relationships observed in the present research.

### 2.2 Dark triad personality traits

The concept of the Dark Triad was first introduced by Paulhus and Williams (2002) to describe three personality traits characterized by manipulative, self-centered and socially negative tendencies. This construct comprises three dimensions—narcissism, Machiavellianism and psychopathy—each representing a different aspect of the dark personality (Birkás et al., 2016). Individuals possessing these traits exhibit conflictual interpersonal orientations characterized by low empathy, a tendency to manipulate others, and self-centered motivations (Thomaes et al., 2017). Although conceptually distinct, these three traits share common characteristics such as emotional coldness, self-interest, self-centeredness and aggression (Paulhus and Williams, 2002). Consequently, the DTP are associated not only with individuals' social relationships but also with various aspects of consumer behavior (Karampournioti et al., 2018).

Narcissism is characterized by an individual's perception of their own superiority, a belief in their own exceptionalism, and a strong need for admiration (Steiner et al., 2021). In contrast, Machiavellianism refers to the tendency to strategically influence and manipulate others to achieve personal gain (Jauk and Dieterich, 2019; Piper et al., 2024). Psychopathy, meanwhile, is defined by low empathy, high impulsivity and a tendency to disregard social norms (Gray et al., 2019). When these traits are considered together, they are associated with low self-regulation, high sensation-seeking and a strong preference for immediate gratification in individuals; this, in turn, increases the likelihood of engaging in impulsive and uncontrolled consumption behaviors (Yousaf et al., 2022; Piper et al., 2024).

From the perspective of consumer behavior, the DTP can be viewed not only as relatively stable personality traits associated with behavioral outcomes, but also as enduring individual tendencies that shape how consumers interact in digital environments (Khan et al., 2025). This approach supports the conceptualization of the DTP as trait-like constructs representing relatively stable individual tendencies. Consistent with the LST approach adopted as the conceptual interpretative framework in the present study, it is proposed that these relatively stable tendencies may be associated with OIB via context-sensitive psychological processes emerging in digital environments. Rather than proposing a temporal or causal sequence, this conceptualization provides a theoretical framework for interpreting the statistical relationships examined in this study.

Previous studies have shown that there are significant relationships between the DTP and various consumer behaviors. However, most of these studies assume that personality traits directly influence consumer behavior. Consequently, less attention has been paid to the context-sensitive psychological processes through which these relatively stable personality characteristics may become associated with consumer behavior in digital environments.

### 2.3 Social media addiction

It is not sufficient to explain the relationship between the ‘Dark Triad' personality traits and OIB solely through direct relationships; additional psychological processes that shape this relationship must also be taken into account. In this context, SMA is attracting increasing attention as an important psychological construct that may help explain the relationship between personality traits and OIB within the context of digital environments. SMA is characterized by an individual's excessive mental focus on social media use, difficulty in controlling their use, and, as a result, the emergence of negative consequences in daily life functioning (Andreassen et al., 2017; Bottaro et al., 2025).

In the literature, SMA is conceptualized as both a psychological and a context-sensitive construct. Andreassen et al. (2017) and Andreassen and Pallesen (2014) define SMA as a psychological construct characterized by a loss of control over social media use, excessive mental preoccupation and functional impairment. In contrast, Kircaburun et al. (2019) and Demircioglu and Göncü Köse (2021) argue that SMA reflects not only individual predispositions but also contextual effects arising from environmental stimuli and the dynamics of digital interaction. Similarly, Wegmann et al. (2020) and Montag et al. (2024) note that factors such as the intensity of social media use, content characteristics and the level of interaction are associated with changes in the experiences and behaviors individuals exhibit in different situations; consequently, they suggest that SMA is a dynamic and context-sensitive construct rather than a fixed personality trait.

SMA is related to both individuals' psychological characteristics and the effects of the digital environment. For this reason, this study examines SMA within the framework of the LST approach. In this approach, SMA is viewed as a state-like psychological process that may help explain the relationship between relatively stable personality traits and impulsive online purchasing. However, previous studies have not reached a consensus on how SMA should be addressed. While some researchers view it as an individual tendency, others see it as a psychological process arising from the influence of the digital environment. These differing perspectives constitute one of the reasons why SMA is addressed within the framework of the LST approach in this study.

### 2.4 Online impulsive buying

Consumers do not always plan every purchase they make. Some buying decisions occur spontaneously in response to immediate desires, emotions, or cues in the shopping environment. Such purchases are commonly referred to as impulsive buying (Gulfraz et al., 2022). Research has traditionally explained this behavior by emphasizing environmental influences, particularly promotional campaigns, product recommendations, and the convenience associated with online shopping (Kathuria and Bakshi, 2024). Yet these factors do not fully explain why some consumers are more likely than others to make impulsive purchases. For this reason, increasing attention has been given to individual psychological characteristics and internal processes (Chan et al., 2017). In online settings, impulsive buying refers to purchases made without prior planning or purchase intention (Anoop and Rahman, 2025).

One reason why OIB has attracted growing attention is that its outcomes are not exclusively positive. While unplanned purchases may provide immediate pleasure, they have also been associated with several undesirable consequences, including lower wellbeing and sustainability-related concerns (Iyer et al., 2020; Olsen et al., 2022). Self-regulation theory offers a useful perspective for understanding this pattern. The theory suggests that impulsive buying becomes more likely when cognitive control is no longer sufficient to regulate pleasure-seeking motives. As self-regulatory capacity weakens, resisting unplanned purchases becomes increasingly difficult (Verplanken and Sato, 2011; Vihari et al., 2022).

In this study, OIB is considered not merely as an individual consumption preference, but as a multidimensional phenomenon that can be explained by considering psychological and behavioral processes together. From this perspective, personality traits are among the fundamental individual differences that influence consumers' decision-making tendencies and purchasing behavior (Ajzen, 1991; Corr and Matthews, 2009). Accordingly, based on the LST approach adopted in this study, OIB is conceptualized as the behavioral outcome of the proposed trait–state–behavior framework, which integrates relatively stable personality traits with context-sensitive psychological processes. Although existing studies have identified numerous antecedents of OIB behavior, the majority of these studies have focused either on environmental stimuli or individual traits. In contrast, conceptual frameworks that can bring these two perspectives together within a single explanatory process have received relatively limited attention in the literature.

## 3 Development of research hypotheses

The relationship between the DTP and SMA can be better explained by the LST approach. According to the LST theory, individual differences are shaped by both relatively stable personality traits and situation-dependent effects (Steyer et al., 2015). This approach was adopted in the present study. The DTP traits were treated as relatively stable personality traits. SMA was considered a situation-dependent psychological process related to individuals' experiences in digital environments.

The DTP is characterized by common traits such as manipulative behavior, self-centeredness, low empathy, and pleasure seeking (Paulhus and Williams, 2002). These traits may influence individuals' social relationships and their use of social media. Research shows that individuals with higher levels of DTP use social media more frequently. They often use social media to gain social approval, present themselves, and achieve personal goals. For example, Machiavellian individuals manage their social media interactions in a more planned and strategic way (Carré et al., 2020; Liao et al., 2025). Narcissistic individuals, on the other hand, are more active on social media to present themselves and gain admiration (Casale and Banchi, 2020; Nguyen et al., 2025). Psychopathy is associated with higher impulsivity and lower self-regulation. Individuals with these characteristics are more likely to use social media in an uncontrolled way (Jauk and Dieterich, 2019).

The LST approach adopted in this study suggests that relatively stable personality traits may be associated with differences in social media use. This effect may become more pronounced because social media platforms offer constant feedback, social validation, and personalized content. Indeed, previous studies have shown positive associations between the DTP and problematic and addictive levels of social media and internet use (Jauk and Dieterich, 2019; Turan et al., 2024; Özsoy et al., 2026). Therefore, this study hypothesizes that the DTP are positively associated with SMA. Accordingly, the following hypothesis is proposed:

**H1**. DTP traits (a: narcissism, b: Machiavellianism and c: psychopathy) have a positive effect on SMA.

It has been noted that more intensive interaction with social media leads to changes in consumer behavior by increasing individuals' exposure to marketing stimuli in digital environments (Dwivedi et al., 2021). The personalized content, instant notifications and opportunities for constant interaction offered by social media platforms capture users' attention and create conditions that make it difficult to maintain self-regulation. In such situations, consumers tend to adopt faster decision-making processes based on less deliberation; it is stated that these processes are associated with impulsive purchasing tendencies (LaRose and Eastin, 2002; Mundel et al., 2024). Similarly, previous studies have shown that SMA is associated with a reduction in self-regulation and a strong preference for immediate gratification, and that these characteristics in turn increase impulsive consumption behaviors (Liu et al., 2025). Furthermore, it is noted that excessive social media use is associated with negative psychological experiences such as stress, anxiety and low self-esteem; and that some individuals turn to shopping behavior in order to cope with or compensate for these negative experiences (Roberts and David, 2020; Maccarrone-Eaglen and Schofield, 2023). When these findings are considered together, it can be argued that SMA is a significant psychological process associated with OIB, both through a weakening of cognitive control and an increased need for emotional regulation.

In line with the LST approach adopted in this study, SMA is conceptualized as a context-sensitive, state-like psychological process that is statistically associated with the relationship between relatively stable personality traits and OIB under the conditions of digital environments. From this perspective, the constant stimuli, personalized content and instant purchasing opportunities offered by social media platforms may strengthen the statistical relationship between individuals' relatively stable personality traits and OIB; however, this should not be interpreted as a direct causal process. In line with these theoretical grounds and previous empirical findings, the following hypothesis has been developed:

**H2**. SMA has a positive effect on OIB behavior.

Explaining the relationship between the DTP and OIB solely through direct effects is insufficient. Previous research indicates that this relationship may also be shaped through psychological processes such as impulsivity, reduced self-regulation, hedonic orientation and self-presentation (Zhu et al., 2023; Khan et al., 2025). In this context, SMA is considered an important psychological process that may help explain the relationship between the DTP and OIB.

A growing body of research has revealed positive associations between the DTP and SMA (Kircaburun et al., 2019; Demircioglu and Göncü Köse, 2021; Liao et al., 2025). It has been observed that individuals with high levels of narcissism, Machiavellianism and psychopathy use social media for different psychological purposes. While narcissistic individuals tend to use social media to present themselves and gain social approval (Necula, 2020; Malesza and Kalinowski, 2021), Machiavellian individuals make greater use of these platforms to engage in strategic interactions and manage their public image (Arli et al., 2019; Blair et al., 2022). In contrast, psychopathic traits are associated with more impulsive and less controlled forms of social media use (Lee, 2019; Siah et al., 2021). Despite these differences in motivation, previous studies have consistently shown positive associations between the DTP and problematic or addictive levels of social media use (Kircaburun et al., 2019; Demircioglu and Göncü Köse, 2021; Liao et al., 2025). Furthermore, it is stated that SMA is associated with greater exposure to marketing stimuli, a reduction in cognitive control and a strengthening of tendencies toward impulsive decision-making; these processes are also linked to OIB behavior (Verhagen and Van Dolen, 2011; Mundel et al., 2024).

In line with the LST approach adopted in this study, the DTP are conceptualized as relatively stable trait-like constructs, while SMA is conceptualized as a context-sensitive state-like psychological process. Within this conceptual framework, it is expected that SMA will be statistically associated with the relationship between the DTP and OIB. Accordingly, the following mediating hypotheses have been developed:

**H3**. SMA mediates the effect of the DTP (a: narcissism, b: Machiavellianism and c: psychopathy) on OIB behavior.

**Figure 1:**
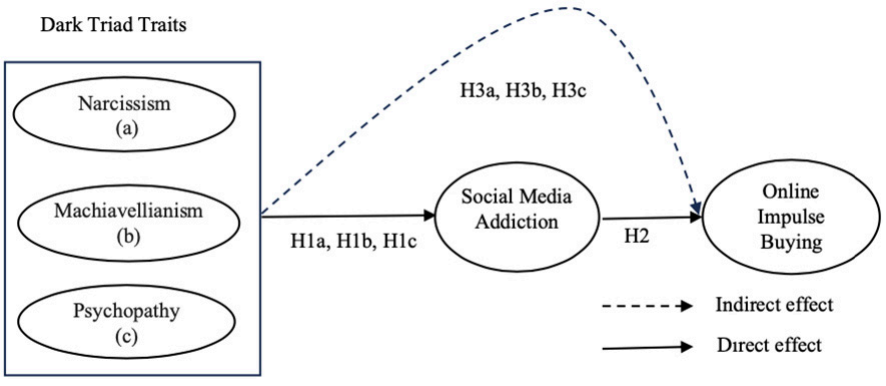
Conceptual model.

The conceptual research model developed in line with the hypotheses outlined above is presented in Figure 1.

## 4 Research methodology

### 4.1 Measures

In this study, a structured questionnaire was used as the data collection tool. The questionnaire was developed following a comprehensive review of the relevant literature, and all constructs included in the research model were measured using scales whose validity and reliability had previously been established. The final measurement tool consists of a total of 23 items.

The ‘Dark Triad' personality traits were measured using the validated Turkish version of the Dirty Dozen scale, developed by Jonason and Webster (2010) and adapted into Turkish by Yaşlioglu and Atilgan (2018). The scale comprises three dimensions (Machiavellianism, narcissism and psychopathy) with each dimension measured by four items. OIB was measured using a five-item scale adapted to the online shopping context by Rizqi Febriandika et al. (2024), based on the Impulsive Buying Behavior scale developed by Rook and Fisher (1995). SMA, on the other hand, was measured using a six-item scale adapted by Coker et al. (2025) based on the Facebook Addiction Scale developed by Andreassen et al. (2012).

As the original versions of the OIB and SMA scales were developed in English, both scales were translated into Turkish using the translation–back translation method. Following the translation process, the translated items were reviewed by subject matter experts to ensure conceptual equivalence and linguistic appropriateness. The wording of the items was adapted to the context of this study while preserving their original conceptual meanings. All constructs were measured using a five-point Likert scale ranging from 1 = strongly disagree to 5 = strongly agree, and responses were numerically coded prior to data analysis. The measurement items used in this study are presented in Table 1. For the mediation analyses, the mean score of the items corresponding to each construct was calculated. Higher scores indicate higher levels of the respective construct, and these mean scores were used in the subsequent analyses.

**Table 1:** Scale items and descriptive statistics.

Variables and scale items		***n*** = **413**			
		Mean	*S.D*.	Kurt.	Skew.
Narcissism (NARS)					
NARS1	I may desire others to admire me.	2.23	1.33	0.718	−0.842
NARS2	I may want others to pay attention to me.	1.85	1.22	1.460	1.026
NARS3	I tend to seek prestige and status.	2.03	1.23	1.073	0.020
NARS4	I tend to expect preferential treatment from others.	1.91	1.22	1.258	0.424
Machiavellianism (MACH)					
MACH1	I can manipulate people to get what I want.	2.17	1.25	0.846	−0.447
MACH2	I have resorted to deception and lying to achieve my goals.	2.01	1.28	1.132	0.082
MACH3	I may flatter people to get what I want.	2.44	1.33	0.515	−1.058
MACH4	I may use people to achieve my own goals.	2.03	1.22	1.108	0.149
Psychopathy (PSYC)					
PSYC1	I tend not to feel guilty about the things I have done.	2.74	1.30	0.063	−1.303
PSYC2	Whether my behavior is moral or not does not concern me.	3.46	1.25	−0.743	−0.610
PSYC3	I can be indifferent or insensitive toward others.	3.17	1.21	−0.412	−0.920
PSYC4	I may behave in a condescending manner toward others.	2.58	1.20	0.256	−1.063
Social Media Addiction (SMA)					
SMA1	Thinking about social media and planning how to use it takes up a lot of my time.	2.33	1.16	0.717	−0.457
SMA2	I feel the need to spend more and more time on social media.	2.68	1.23	0.292	−1.086
SMA3	I use social media to forget my personal problems.	3.20	1.24	−0.354	−1.058
SMA4	I try to reduce the time I spend on social media, but I fail.	2.76	1.26	0.270	−1.113
SMA5	I feel restless when my social media use is restricted.	2.89	1.24	−0.029	−1.167
SMA6	I spend so much time on social media that it negatively affects my work (or studies).	2.34	1.22	0.673	−0.634
Online Impulsive Buying (OIB)					
OIB1	I often make spontaneous purchases online.	2.55	1.19	0.355	−1.097
OIB2	The phrase “Just do it” describes my online shopping style.	2.23	1.22	0.859	−0.252
OIB3	I often shop online without thinking.	2.18	1.22	0.985	−0.035
OIB4	The phrase “I saw it, I bought it” describes me in online shopping.	2.38	1.26	0.595	−0.847
OIB5	The phrase “Buy now, think later” perfectly reflects my online shopping behavior.	2.20	1.21	0.881	−0.303

Table 1 presents the mean, standard deviation, kurtosis and skewness values of the scale statements. According to the findings, the skewness values of all items ranged between −1.303 and 1.026, while the kurtosis values ranged between −0.743 and 1.460. As all values were within the ±1.96 criterion proposed by Kim (2013), the assumption of normality was considered to be satisfied. Accordingly, it was concluded that it is appropriate to use parametric analysis methods to test the research hypotheses.

### 4.2 Sample selection, data collection and sample characteristics

The study's target population consists of individuals living in Turkey who are 18 years of age or older, actively use social media, and have previously made online purchases. These criteria were established to ensure that participants have the necessary experience to answer questions related to SMA and OIB. Convenience sampling was used in the study. Data were collected between June 25 and 30, 2025, via an online survey created using Google Forms. Support was provided by the company Bilimsel Anketler to reach participants. The company used a machine learning-supported digital targeting system to identify participants who met the study criteria. The survey link was distributed through Google Ads, Meta Ads, social media platforms, and online forums. The target group consisted of active social media users who had previously made online purchases. Before the survey started, the purpose of the study was explained to the participants, and their informed consent was obtained. Participation in the study was completely voluntary. No personal information that could identify the participants was collected. Each participant completed the survey individually. Participants were also informed that a gift card raffle would be held after the data collection process was completed.

Initially, 424 questionnaires were collected. Before the data were sent to the researchers, they were checked by the survey company. Standard data quality checks were carried out at this stage. As a result, 11 questionnaires were removed from the dataset. These questionnaires were excluded because they were incomplete, did not meet the study's participation criteria, or contained low-quality responses. An example of a low-quality response is selecting the same answer for every question.

Thus, the analyses were conducted using 413 valid surveys. Consequently, only questionnaires that satisfied the inclusion criteria and data quality standards were retained, resulting in a final dataset of 413 valid questionnaires, which were included in the final analyses. An a priori power analysis was conducted using the G^*^Power 3.1 program to determine the required minimum sample size. As there is no direct option in G^*^Power for covariance-based structural equation modeling (CB-SEM), the structural equation multiple regression approach, which includes the largest number of predictors, was used for the assessment. Accordingly, the analysis was carried out using the F-tests family and the Linear multiple regression: Fixed model, R^2^ deviation from zero statistical test. Assuming a medium effect size (f^2^ = 0.15), a significance level of α = 0.05, a statistical power of 0.95 and four predictors, the required minimum sample size was calculated as 129 participants. Therefore, the 413-person sample used in this study was deemed sufficient to test the proposed structural model.

When the demographic profile in Table 2 is examined, the sample is largely composed of female participants, who account for 77.97% of the respondents. Although women are overrepresented in the sample, this pattern is consistent with prior survey methodology research showing that women tend to exhibit higher response rates in web-based surveys than men (Becker, 2022; Wu et al., 2022). In terms of age, the majority fall within the 26–41 range, representing 53.51% of the sample, followed by those aged 18–25, who make up 18.64%. This indicates that the sample mainly consists of young and middle-aged individuals. In terms of daily social media use, participants appear to be quite active. About 45.76% report spending between 1 and 3 h per day on social media, while 31.72% spend between 3 and 6 h. Regarding online shopping behavior, most participants shop relatively infrequently, with 44.55% making purchases once a month or less and 32.93% shopping 2 to 3 times per month. Finally, Instagram clearly stands out as the dominant platform in this sample, with 98.54% of participants reporting that they use it.

**Table 2:** Demographic and socio-economic characteristics of the sample.

Gender	*n*	*%*	Age	*n*	*%*
Female	322	77.97	18-25 age	77	18.64
Male	91	22.03	26-33 age	99	23.97
Social media platforms used^*^	*n*	*%*	34-41 age	122	29.54
Instagram	407	98.54	42-49 age	71	17.19
Facebook	225	54.48	50 - +	44	10.66
X	158	38.26	Monthly online purchases	*n*	*%*
Pinterest	97	23.49	Once or less	184	44.55
Tik Tok	107	25.91	2–3 times	136	32.93
Time spent on social media per Day	*n*	*%*	4–5 times	52	12.59
Less than 1 h	32	7.75	6–7 times	29	7.02
1–3 h	189	45.76	8 times or more	12	2.91
3–6 h	131	31.72			
More than 6 h	61	14.77			

^*^Since multiple responses were allowed, the total percentages may exceed 100%.

## 5 Results

Firstly, a confirmatory factor analysis (CFA) was conducted to assess the validity and reliability of the measurement model. Next, the structural model was analyzed using covariance-based structural equation modeling (CB-SEM) with LISREL 8.80 to test the direct relationships among the variables. The mediating role of SMA in the relationships between the DTP dimensions (narcissism, Machiavellianism, and psychopathy) and OIB was then examined using Hayes's PROCESS macro (Version 4.2) in IBM SPSS Statistics 26. Indirect effects were estimated using 5,000 bootstrap resamples, and their statistical significance was determined based on bias-corrected bootstrap confidence intervals.

### 5.1 Assessment of measurement model

Before testing the research hypotheses, the appropriateness of the measurement model and the reliability and validity coefficients of the variables in the study were examined. In this context, firstly, the internal consistency of the scales was evaluated and Cronbach's Alpha coefficients are given in Table 3. The results obtained reveal that the Cronbach's Alpha values of all scales are sufficiently reliable.

**Table 3:** Goodness of fit statistics for the measurement of CFA and structural model SEM.

Classification	Fit index	CFA model	SEM model	Acceptable values
Chi-square	χ^2^	444.30	507.75	–
Degrees of freedom (df)	df	220	223	–
Absolute fit measurements	χ^2^/df	2.02	2.28	< 5
	GFI	0.92	0.91	>0.90
	AGFI	0.90	0.90	>0.90
	RMSEA	0.053	0.056	< 0.08
Incremental fit measurements	CFI	0.99	0.98	>0.90
	IFI	0.99	0.98	>0.90
	NFI	0.97	0.97	>0.90

Following the reliability analysis, CFA was conducted to test the structural appropriateness of the measurement model. The findings of the analysis in Table 3 show that the goodness of fit indices of the model are within the acceptable and good fit ranges (χ^2^/df = 2.02; RMSEA = 0.053; CFI = 0.99; GFI = 0.92; IFI = 0.99; NFI = 0.97). These results show that the measurement model is compatible with the data.

To evaluate the adequacy of the measurement model, the strength of the relationships between the indicators and their respective constructs was first assessed through factor loadings. As reported in Table 4, all standardized loadings exceed the recommended threshold of 0.60, indicating that the indicators reliably capture their underlying constructs and that the measurement model demonstrates satisfactory indicator reliability (Hair et al., 2022). In order to ensure convergent validity, the composite reliability (CR) coefficients of the scales should be higher than 0.70, the average variance explained (AVE) values should be higher than 0.50, and all CR values should be greater than AVE values (Henseler et al., 2015; Hair et al., 2022). It is seen that the CR values of the study are between 0.822-0.918; AVE values are between 0.501-0.694 and CR values are higher than AVE values (Table 4). In line with these results, it can be said that convergent validity is achieved.

**Table 4:** Construct validity, reliability, and multicollinearity.

Latent construct	Measured variable	Standardized factor loading (CFA) (>0.6)	VIF outer model	Cronbach's Alpha (>0.7)	CR (>0.7)	AVE (>0.5)
Narcissism (NARS)	NARS1	0.65	1.671	0.887	0.892	0.678
	NARS2	0.89	3.419			
	NARS3	0.85	2.869			
	NARS4	0.88	3.301			
Machiavellianism (MACH)	MACH1	0.79	2.303	0.898	0.899	0.691
	MACH2	0.87	3.077			
	MACH3	0.78	2.187			
	MACH4	0.88	3.143			
Psychopathy (PSYC)	PSYC1	0.75	1.769	0.822	0.822	0.537
	PSYC2	0.74	1.812			
	PSYC3	0.72	1.693			
	PSYC4	0.72	1.652			
Social Media Addiction (SMA)	SMA1	0.62	1.433	0.853	0.857	0.501
	SMA2	0.76	1.979			
	SMA3	0.72	1.803			
	SMA4	0.72	1.907			
	SMA5	0.72	1.829			
	SMA6	0.70	1.777			
Online Impulsive Buying (OIB)	OIB1	0.69	1.911	0.917	0.918	0.694
	OIB2	0.83	2.807			
	OIB3	0.90	3.852			
	OIB4	0.86	3.064			
	OIB5	0.87	3.270			

The Fornell and Larcker (1981) criterion was taken as a basis in the examination of discriminant validity. The findings obtained show that the square root of the AVE values of each construct is higher than the correlation values of the related construct with other constructs (Table 5). These findings indicate that the discriminant validity is also provided in the measurement model. As a result, it was determined that the reliability and validity conditions for the measurement model were met, and it was considered appropriate to proceed to the structural model analysis in line with the data obtained.

**Table 5:** Assessment of discriminant validity.

Latent construct	Mean	SD	Correlation matrix				
			SMA	OIB	MACH	NARS	PSYC
**Social Media Addiction (SMA)**	2.70	0.93	**0.707**				
**Online Impulsive Buying (OIB)**	2.30	1.06	0.530	**0.833**			
**MACHiavellianism (MACH)**	2.16	1.11	0.503	0.488	**0.831**		
**Narcissism (NARS)**	2.01	1.08	0.472	0.431	0.626	**0.823**	
**PSYChopathy (PSYC)**	2.98	1.00	0.425	0.316	0.382	0.317	**0.733**

The bold diagonal values are the Average Variance Extracted (AVE) square root.

### 5.2 Evaluation of the structural model

The hypotheses proposed in the research model were analyzed within the scope of CB-SEM. When the goodness-of-fit indicators of the structural model in Table 3 are evaluated, it is seen that the values obtained are generally within the acceptable and good fit ranges (χ^2^/df = 2.28; RMSEA = 0.056; CFI = 0.98; IFI = 0.98; NFI = 0.97; GFI = 0.91; AGFI = 0.90) (Schermelleh-Engel et al., 2003). These results indicate that the model exhibits a structure compatible with the empirical data.

When the direct relationships between the constructs in Table 6 were analyzed, the results show that the DTP dimensions had significant effects on SMA. In this context, the effect of narcissism on SMA is positive and statistically significant (β = 0.24; t = 3.50; *p* < 0.01). Similarly, Machiavellianism has a significant effect on SMA (β = 0.29; *t* = 3.96; *p* < 0.01). Among the three Dark Triad dimensions, psychopathy exhibited the largest standardized path coefficient (β = 0.32), indicating the strongest statistical relationship with SMA (β = 0.32; *t* = 5.52; *p* < 0.01). Accordingly, hypotheses H1a, H1b and H1c were confirmed. When the effect of SMA on OIB behavior is evaluated, it is seen that this relationship is statistically significant and positive (β = 0.61; t = 8.71; p < 0.01). This finding reveals that as the level of SMA increases, individuals' impulsive buying tendencies also increase. Therefore, the H2 hypothesis is supported.

**Table 6:** Evaluation of structural equation model analysis (Direct effect).

Relationships	β	*t*-value	*S.E*.	VIF	Result	*R* ^2^
H1a: NARS = > SMA	0.24	3.50^*^	0.068	1.471	Supported	0.46
H1b: MACH = > SMA	0.29	3.96^*^	0.073	1.657	Supported	
H1c: PSYC = > SMA	0.32	5.52^*^	0.090	1.381	Supported	
H2: SMA = > OIB	0.61	8.71^*^	0.083	1.345	Supported	0.37

p^*^ < 0.01.

When the model's explanatory power is examined, it is found that the DTP traits explain 46% of the variance in SMA (*R*^2^ = 0.46). In addition, the model explained 37% of the variance in OIB (R^2^ = 0.37). These values indicate that the model's explanatory capacity is sufficient. Finally, VIF values were analyzed to assess multicollinearity. The findings show that the VIF values range from 1.345 to 1.657 and remain below the critical threshold (Hair et al., 2022). This reveals that there is no multicollinearity problem in the model.

### 5.3 Results of the bootstrap-based mediation analysis

Bootstrap-based regression analysis was preferred to determine the mediating role of SMA in the effect of each of the narcissism, Machiavellianism and psychopathy dimensions of the DTP traits on OIB. In the study, bootstrap procedure with 5,000 resamples was applied, and it was concluded that the mediation effect was statistically significant when confidence intervals did not include zero within the 95% confidence interval.

The findings presented in Table 7 reveal the mediating role of SMA in the relationship between DTP and OIB behavior. The effect of narcissism on SMA is positive and statistically significant (β = 0.405; 95% CI [0.332; 0.479]; t = 10.856; p < 0.001). When SMA was included in the model, the direct effect of narcissism on OIB decreased but remained significant (c = 0.421; *p* < 0.01; c' = 0.227; *p* < 0.01). In addition, the effect of SMA on OIB was strong and significant (β = 0.479; 95% CI [0.376; 0.582]; t = 9.117, *p* < 0.001). Bootstrap findings show that the indirect effect is significant (β = 0.194; 95% CI [0.1381; 0.2610]) and these findings are consistent with a statistically significant partial indirect effect.

**Table 7:** Bootstrap-based regression analysis results (Indirect effect).

Effects	ß	*SE*	*t*	*p*-value	LLCI	ULCI
NARS → SMA (a)	0.405	0.037	10.856	0.000	0.332	0.479
SMA → *OIB* (b)	0.479	0.053	9.117	0.000	0.376	0.582
MACH → SMA (a)	0.421	0.036	11.802	0.000	0.351	0.491
SMA → *OIB* (b)	0.434	0.053	8.252	0.000	0.331	0.538
PSYC → SMA (a)	0.394	0.041	9.532	0.000	0.313	0.475
SMA → *OIB* (b)	0.551	0.052	10.516	0.000	0.448	0.636
Direct effect						
NARS → *OIB* (c')	0.227	0.045	5.044	0.000	0.139	0.316
MACH → *OIB* (c')	0.282	0.044	6.399	0.000	0.195	0.368
PSYC → *OIB* (c')	0.116	0.048	2.392	0.017	0.021	0.211
Total effect (c)						
NARS → *OIB*	0.421	0.034	9.681	0.000	0.336	0.507
MACH → *OIB*	0.465	0.041	11.320	0.000	0.384	0.545
PSYC → *OIB*	0.333	0.049	6.742	0.000	0.236	0.430
Indirect effects	ß	BootSE	BootLLCI	BootULCI		Results
H3a: NARS → SMA → *OIB*	0.194	0.0313	0.1381	0.2610		Partial mediation
H3b: MACH → SMA → *OIB*	0.183	0.031	0.1265	0.2451		Partial mediation
H3c: PSYC → SMA → *OIB*	0.217	0.033	0.1537	0.2859		Partial mediation

The findings obtained in terms of Machiavellianism show that this personality trait showed a statistically significant indirect effect through SMA. The effect of Machiavellianism on SMA is significant (β = 0.421; *t* = 11.802; *p* < 0.001), suggesting that this variable plays an important role in shaping behaviors in the digital environment. When SMA is included in the model, although there is a decrease in the effect of Machiavellianism on OIB, this effect maintains its statistical significance (c = 0.465; p < 0.01; c' = 0.282; *p* < 0.01). In addition, SMA remained significantly associated with OIB (β = 0.434; t = 8.252; *p* < 0.001). The indirect effect coefficient was significant (β = 0.183; 95% CI [0.1265; 0.2451]), indicating a statistically significant indirect effect through SMA.

The findings regarding the psychopathy dimension present a relatively different effect pattern. Although the effect of psychopathy on SMA is positive and significant (β = 0.394; t = 9.532; *p* < 0.001), it is noteworthy that its direct effect on OIB is more limited compared to other personality dimensions (β = 0.116; t = 2.392; *p* < 0.05). When SMA was added to the model, the direct effect decreased but remained significant (c = 0.333, *p* < 0.01; c' = 0.116; *p* < 0.05). However, SMA emerges as a strong determinant in this model as well (β = 0.551; t = 10.516; *p* < 0.001). Bootstrap analysis results (β = 0.217; 95% CI [0.1537; 0.2859]) suggest that SMA also has a partial mediating role in the relationship between psychopathy and OIB. In general, it is understood that SMA plays a significant and partial mediating role in the relationship between DTP and OIB behavior. In this context, H3a, H3b and H3c hypotheses were supported.

## 6 Evaluation of findings

The findings of this study indicate that there are statistically significant relationships between the DTP, SMA and OIB. Drawing on the LST framework, the DTP are interpreted as relatively stable individual characteristics, while SMA is regarded as a state-like psychological process associated with these personality traits and OIB. Rather than empirically testing a formal LST model, this study uses the LST theory as a conceptual framework to interpret the observed statistical relationships. Accordingly, the findings suggest that the relationship between the DTP and OIB can be explained via an indirect process involving SMA.

Firstly, it was found that narcissism is positively associated with SMA. This finding is consistent with previous studies (Marino et al., 2021; Giancola et al., 2025; Nguyen et al., 2025). The strong need for admiration and external validation exhibited by narcissistic individuals has long been recognized as one of the key motivations for social media use. However, the current findings extend previous evidence by showing that these motivations are associated not only with more frequent social media use but also with problematic forms of use resembling addictive tendencies. Likes, comments, and indicators of social approval on social media platforms may reinforce these trends over time. This situation may contribute to using social media platforms more intensively. Previous studies have also shown that social approval is associated with problematic social media use (Andreassen et al., 2017; Servidio et al., 2021; Vaid and Harari, 2021). The findings of this study support these previous studies. In other words, as narcissistic personality traits increase, so does problematic social media use.

Similarly, Machiavellianism was found to be positively associated with SMA, a result that supports previous research (Hussain et al., 2021; Tang et al., 2022). However, the current findings suggest that this relationship may reflect not only intensive social media use but also the functional and strategic use of social media. It has been shown that individuals with high levels of Machiavellianism use social media not only for emotional regulation (Gómez-Leal et al., 2023), but also to manage interpersonal relationships, create impressions and influence others (Wang et al., 2026). Consequently, social media may function as an instrumental interpersonal environment rather than merely a source of entertainment or pleasure (Abell and Brewer, 2014). Such strategic use may be associated with more intensive platform use and, consequently, higher levels of problematic social media use. Although the cross-sectional design of this study does not permit inferences regarding causality, the findings are consistent with previous literature indicating that Machiavellian tendencies are positively associated with problematic use of social media platforms.

Similarly, psychopathy has been found to be positively associated with SMA. This finding is consistent with previous studies (Cerniglia et al., 2019; Chung et al., 2019; Sparavec et al., 2022). This relationship can be explained by the relatively anonymous, less regulated and socially permissive nature of online environments. Such environments may enable individuals with psychopathic tendencies to more readily display traits such as manipulation, superficial interpersonal relationships and dominance (Demircioglu and Göncü Köse, 2021; Elhami Athar, 2025). Furthermore, impulsivity and sensation-seeking—which are core characteristics of psychopathy—may encourage greater engagement with highly stimulating digital content (Fox and Rooney, 2015). When these findings are considered together, it becomes clear that all three dimensions of the Dark Triad are positively associated with SMA; however, these associations may arise through different psychological processes.

The findings also demonstrate a positive correlation between SMA and OIB. This result is consistent with previous studies that have shown social media platforms to be not only communication channels but also digital environments where marketing stimuli, social comparison processes, and user interactions are associated with impulsive buying decisions (Habib and Almamy, 2025). Continuous exposure to digital content, personalized recommendations, and influencer marketing can increase consumers' tendency to make spontaneous purchasing decisions. Previous research has shown that fear of missing out (FOMO) (Bazi et al., 2026) and idealized online self-presentations (Miranda et al., 2023) are associated with stronger impulsive buying tendencies. Similarly, it has been noted that excessive social media use is linked to decreased self-control, which can increase consumers' tendency to seek instant gratification through impulsive purchases (Liu et al., 2025). Accordingly, the current findings support previous literature showing that individuals with high levels of SMA also exhibit more OIB behavior.

Overall, the statistically significant indirect effects obtained in this study suggest that SMA showed statistically significant indirect effects linking the DTP and OIB. Individuals with a higher reward-seeking tendency and relatively lower levels of self-control (Malesza and Kalinowski, 2021) reported higher levels of SMA; and SMA was found to be positively correlated with OIB. These results are consistent with previous studies showing that individuals who engage heavily with social media are constantly exposed to marketing stimuli, social comparison processes, and social validation cues, and that these elements are associated with impulsive buying tendencies (Chung et al., 2019; Mundel et al., 2024; Özsoy et al., 2026). The current findings suggest that SMA can be interpreted not as a causal transmission mechanism, but rather as a statistically significant indirect process linking relatively stable personality traits to OIB behavior. However, other explanations are also possible. For example, individuals who frequently engage in OIB may start following more brands, influencers, and shopping-related accounts over time. This may lead to greater use of social media. Longitudinal studies are needed to determine the order of these relationships over time. This finding is also consistent with Self-Control Theory, which suggests that impulsive behavior increases as self-control decreases (Baumeister, 2002).

This study shows that the relationship between the DTP and OIB is not only direct. Psychological processes that emerge in digital environments also play a role in this relationship. Therefore, it is not enough to focus only on personality traits when explaining OIB. The psychological processes that arise in digital environments should also be considered. The findings show that OIB is associated with both personality traits and the psychological processes that occur during social media use. For this reason, it is more appropriate to consider these two factors together. Another finding is related to SMA. Previous studies have mostly treated SMA as an outcome of personality traits or as a cause of different behaviors. In the present study, however, SMA was considered a psychological process that plays a role in the relationship between personality traits and OIB. This finding suggests that SMA may be a distinct psychological process related to consumer behavior.

The results are also consistent with the LST approach. According to this approach, personality traits are more enduring. SMA, on the other hand, is a situational psychological process. The LST approach was not directly tested in this study. Nevertheless, the findings suggest that this approach provides a useful conceptual framework for interpreting the relationships among the DTP, SMA, and OIB. The significance of the indirect effects also supports this assessment.

### 6.1 Theoretical contributions

The findings suggest that the relationship between the DTP and OIB is statistically associated with significant indirect effects involving SMA. The findings indicate that the relationship between personality traits and OIB does not arise solely through direct relationships, but can also be explained through context-sensitive psychological processes such as SMA. Furthermore, rather than reinterpreting the LST theory, the study demonstrates how concepts derived from the LST framework can be applied as a conceptual lens for understanding the statistical relationships between personality traits, SMA and OIB. Accordingly, the findings suggest that relatively stable personality traits may be associated with state-like psychological processes, and that these processes are, in turn, associated with OIB. Finally, SMA has been conceptualized not as a fixed individual trait, but as a state-like psychological process representing a statistically significant indirect pathway between individual tendencies and OIB. This perspective contributes to a more process-oriented and holistic understanding of OIB behavior.

### 6.2 Managerial implications

OIB should be considered not only as a business opportunity but also as a potential negative outcome of consumption. Because SMA is associated with this behavior, some consumers may be more vulnerable to impulsive buying than others. This makes responsible marketing an important issue for businesses. Businesses should avoid marketing practices that create unnecessary pressure to buy. Frequent notifications, urgency messages, and fear-of-missing-out (FOMO) appeals may increase sales in the short term, but they can also reduce consumer trust over time. Communication strategies that encourage informed purchasing decisions are likely to be more beneficial in the long run. Social media and e-commerce platforms can also help users control their purchasing behavior. Spending limits, time reminders, and short waiting periods before completing a purchase may reduce impulsive buying. Businesses should also be careful when targeting vulnerable consumers. Encouraging excessive consumption may improve short-term sales, but it can also harm consumer wellbeing and damage brand reputation over time.

### 6.3 Limitations and future research

This study has several limitations. First, the cross-sectional design does not allow the temporal sequence among the DTP, SMA, and OIB to be established. Although the proposed mediation model is theoretically grounded and statistically supported, the findings should not be interpreted as evidence of causal mediation, and alternative causal explanations cannot be excluded. Second, the use of convenience sampling from a single-country sample limits the generalizability of the findings. Third, the model focused exclusively on SMA as the mediating mechanism, which restricts the explanatory scope of the study.

Future research should employ longitudinal or experimental designs to examine the causal and temporal dynamics of the trait–state–behavior process proposed within the LST framework. Cross-cultural studies would help assess the generalizability of the findings across different contexts. In addition, incorporating other state-like mechanisms, such as Fear of Missing Out (FOMO), self-control, or emotional states, could provide a more comprehensive explanation of how personality traits become statistically associated with OIB through state-like psychological processes. Finally, future studies may explore whether the proposed relationships vary across different product categories, social media platforms (e.g., Instagram and TikTok), or after controlling for demographic characteristics.

## Data Availability

The raw data supporting the conclusions of this article will be made available by the authors, without undue reservation.
